# Psychodynamic case formulations without technical language: a reliability study

**DOI:** 10.1186/s40359-019-0337-5

**Published:** 2019-10-24

**Authors:** Øystein Sørbye, Hanne-Sofie J. Dahl, Tracy D. Eells, Svein Amlo, Anne Grete Hersoug, Unn K. Haukvik, Cecilie B. Hartberg, Per Andreas Høglend, Randi Ulberg

**Affiliations:** 1 Division of Mental Health and Addiction, Oslo University Hospital, Oslo, Norway; 2 Division of Mental Health and Addiction, Vestfold Hospital Trust, PO Box 2168, 3103 Tønsberg, Norway; 3 Department of Psychiatry and Behavioral Sciences, University of Louisville, Louisville, KY USA; 4 Billingstad, Norway; 5 Division of Mental Health and Addiction, University of Oslo, PO Box 85, 0319 Vinderen, Norway; 6 Department of Psychiatry, Diakonhjemmet Hospital, Diakonveien 12, 0370 Oslo, Norway

**Keywords:** Case formulations, Psychodynamic, Reliability

## Abstract

**Background:**

To bridge the gap between symptoms and treatment, constructing case formulations is essential for clinicians. Limited scientific value has been attributed to case formulations because of problems with quality, reliability, and validity. For understanding, communication, and treatment planning beyond each specific clinician-patient dyad, a case formulation must convey valid information concerning the patient, as well as being a reliable source of information regardless of the clinician’s theoretical orientation. The first aim of the present study is to explore the completeness of unstructured psychodynamic formulations, according to four components outlined in the Case Formulation Content Coding Method (CFCCM). The second aim is to estimate the reliability of independent formulations and their components, using similarity ratings of matched versus mismatched cases.

**Methods:**

This study explores psychodynamic case formulations as made by two or more experienced clinicians after listening to an evaluation interview. The clinicians structured the formulations freely, with the sole constraint that technical, theory-laden terminology should be avoided. The formulations were decomposed into components after all formulations had been written.

**Results:**

The results indicated that most formulations were adequately comprehensive, and that overall reliability of the formulations was high (> 0.70) for both experienced and inexperienced clinician raters, although the lower bound reliability estimate of the formulation component deemed most difficult to rate - inferred mechanisms - was marginal, 0.61.

**Conclusions:**

These results were achieved on case formulations made by experienced clinicians using simple experience-near language and minimizing technical concepts, which indicate a communicative quality in the formulations that make them clinically sound.

**Trial registration:**

linicalTrials.gov Identifier: NCT00423462. 10.1007/s00432-018-2781-7., January 18, 2007.

## Background

Constructing an adequate case formulation is broadly recognized as a core competency for clinicians [1] and a central capacity required to pass the certifying examinations of the American Board of Psychiatry & Neurology [2]. A case formulation is defined as a set of hypotheses about the causes, precipitants and maintaining factors of a patient’s psychological, interpersonal and behavioral problems [3–5]. The primary function of case formulations is to provide a “map” that guides the clinicians in practice and should differentiate what the clinician and patient see as essential from what is secondary or not relevant. There is a wide array of models for making case formulations, from theoretical-specific [6] to trans-theoretical models [7]. A case formulation, regardless of model, is intended to give meaning and context to the chosen intervention whether it is a certain kind of individual psychotherapy, medication management, group therapy, residential treatment, etc. According to Horowitz [8], it fills “a gap that otherwise would exist between diagnosis and treatment” (p. IX). Specifically, board-certified psychiatrists in the United States are expected “to develop and document an integrative case formulation that includes neurobiological, phenomenological, psychological and sociocultural issues involved in diagnosis and management” [9].

While our primary focus is on case formulation in a psychotherapeutic context as practiced by psychiatrists, clinical case formulation can be useful across many mental health disciplines – including social work and psychology - and in multiple types of clinical practice, including medication management. For example, Tasman [10] observed that treatment adherence in pharmacotherapy can be enhanced by conducting a case formulation prior to prescribing. While each discipline and practice may require unique information elements in a formulation, some elements are common to all disciplines, for example, a problem list and an explanatory mechanism that accounts for symptoms and problems. Some definitions of case formulation include an explicit treatment plan, others do not. The treatment plan may be based on the formulation, but not part of it.

Despite the widely acknowledged importance and value of case formulation in clinical settings, formulation has had limited scientific impact because of problems with quality, reliability and undetermined validity [11]. With regard to quality, evidence suggests that the skills necessary to make a case formulation are difficult to acquire [12]. Kuyken and colleagues [13] measured the quality of case formulations by 115 mental health professionals. Only 44% were deemed “good enough”. Eells and colleagues [14] evaluated 56 intake formulations from an outpatient clinic. Ninety-five percent contained descriptive information, but less than half addressed hypothesized predisposing life events and/or inferred psychological mechanisms, which are necessary in a proper case formulation. Comparable results were obtained in the evaluation of biopsychosocial formulations developed by psychiatry residents [15].

Within the psychodynamic tradition, psychoanalysts have tended to conceptualize the dynamics of a given case based on their own theoretical positions, often in rather abstract meta-psychological terms, which had limited communicative and scientific value [16, 17]. Seitz [18] described how a group of psychoanalysts failed to arrive at consensus formulations of cases. He noted that the judges applied different levels of inference when interpreting the clinical data, which led the group to an impasse as to what was centrally important. The formulation method used in this study was based on Malan’s overall case formulation system [19]. Malan never formally tested the reliability of his method. A basic prerequisite for scientific progress in this area is a certain level of agreement among clinicians about case formulations. In an early review, Barber and Crits-Christoph [20] found that structured psychodynamic case formulations are more likely to be reliable. Garb [21] also concluded that inter-rater reliability of structured psychodynamic formulation methods is good if clinicians share the same theoretical orientation and the formulations compared are decomposed into separate components. So far, only structured methods, breaking the formulations down into components and using standard language, have achieved acceptable to good reliability [4, 13, 20–22]. The Case Formulation Content Coding Method (CFCCM) [3, 4, 14] is an example of a structured model. The CFCCM is a method to categorize information clinicians use when conceptualizing a patient. One CFCCM task is to segment a formulation into one of four content areas that are described in most models of case formulations. The main content areas are: (1) symptoms and problems (2) precipitating stressors, (3) predisposing life events, and (4) an explanatory mechanism that links the preceding categories together and offers an explanation of the precipitants and maintaining influences of the individual’s problems. In general, the primary task of content coders is to independently read a written formulation and mark whether a formulation element is present. After completing a set of formulations, the coders compare their codes and discuss disagreement until consensus is reached. The number of content areas addressed in a formulation can serve as a measure of completeness. Interrater reliability can be assessed both for an entire formulation and for each of the four components.

The first aim of the present study is to explore the completeness of unstructured psychodynamic formulations, by decomposing each formulation according to the Case Formulation Content Coding Method (CFCCM) [3, 4, 14], and examine whether or not each formulation contains all components. The second aim is to estimate the reliability of independent formulations and their components, using similarity ratings of matched versus mismatched cases [22].

## Methods

### Sample

The data for this report is based on the First Experimental Study of Transference (FEST) study, a randomized clinical trial designed to study the impact of specific techniques in dynamic psychotherapy [23, 24]. A total of 122 patients were referred to FEST study clinicians by primary care physicians, private specialist practitioners, and public outpatient departments. These patients sought psychotherapy due to depressive disorders, anxiety disorders, personality disorders, and interpersonal problems, as diagnosed using DSM-III-R criteria. The study clinicians assessed the patients for eligibility. Patients with psychosis, bipolar illness, organic mental disorder, substance abuse, and those with other mental health problems that caused long-term inability to work (> 2 years) were also excluded. Each of the 100 participants included in the study gave written informed consent and were then randomly assigned to receive weekly sessions of dynamic psychotherapy for 1 year either with or without transference interpretations [25, 26]. The study protocol was approved by The Regional Ethics Committee, Health Region South East, Norway. The study ID number in www.clinicaltrials.gov is FEST307/95. Patient anonymity has been preserved.

### Semi-structured interviews

The clinical research team consisted of the psychotherapists in the FEST study who were six psychiatrists and one clinical psychologist. They had received their dynamic psychotherapy training at one of four training institutes and had between 10 and 25 years of experience doing psychotherapy. All seven clinicians were in private practice. After taking history and assessment of background variables by the patients’ therapists, one of the clinicians (not the patient’s psychotherapist) conducted a 2-h semi-structured psychodynamic interview, modified from Sifneos [27], and Malan and Osimo [28]. The interview was more open-ended than diagnostic interviews. The interview should focus on behavior, affective experiences, symptoms and problems, and especially current and past maladaptive/adaptive relationships. The interviewer should conduct the interview trying to elucidate warded off material, such as wishes, motives, fears and conflicts, and also help the patient to explore meaningful experiences in detail. The clinician should pay attention to sudden changes in behavior or avoidance of certain topics. The interview was audio recorded.

### Case formulations

A minimum of two, but most often three or more other clinicians from the research team listened to the interview. Subsequently, the clinicians independently wrote a psychodynamic case formulation based on the patient’s clinical history, diagnostic evaluation, and the psychodynamic interview. The formulation should include “a core neurotic conflict” [19] that was seen as central to the patient’s difficulties, and specific stressors to which the patient was assumed vulnerable. Neurotic conflicts indicate how patients repeatedly handle emotional and instinctual impulses in ways that may increase their psychological problems. A treatment plan was not included in the formulation. The clinicians were asked to write the formulations using simple, experience-near terminology with a minimum of technical and theoretical language. Otherwise, they were free to develop the formulations according to their own wish. More than 400 case formulations were written, with an average of 4.2 per patient.

To examine the completeness of the formulations, the first author segmented each of the 425 formulations into four components, according to the Case Formulation Content Coding Method (CFCCM), described earlier. Another evaluator examined the work of the first author and disagreements were discussed until consensus was reached.

### Raters

To assess reliability, we used three pairs of raters. All raters volunteered to be participants in the study. One pair of raters served as clinicians in the FEST study, each of whom had contributed a number of case formulations themselves. They were both psychiatrists and trained psychoanalysts and had more than 20 years of clinical experience. The second pair of raters, a psychiatrist and a specialist in psychology, had not been clinicians in the study. They had their training from a different psychodynamic institute than the fist pair, had long clinical experience, and were psychotherapy supervisors. The third pair of raters was resident psychiatrists, early in their training, with little clinical experience, and barely any knowledge of dynamic psychotherapy. The raters were given a text on a sheet of paper that contained two case formulations and they did not know whether the two formulations were from the same patient (matched pair), or from different patients (mismatched). Each sheet had a random number to ensure blindness on matched or mismatched formulations. The degree of similarity was rated on a Likert scale from 1 to 7. A rating of “7” means that all phrases (thought units) show complete or near complete agreement in meaning. A rating of “1” means that none of the phrases have the same meaning. A score of “4” means that half of the phrases are similar in meaning (For example the same description of the relationship to father, but different or missing concerning mother). The most important content of formulations to rate for similarity should be the patient’s interpersonal relations and personal reactions. Demographic and descriptive information in the text should be regarded as less important. A few times descriptive information indicated a mismatched pair. The raters were advised to disregard this information when evaluating the formulations.

We evaluated the reliability of the whole formulation, as well as that of the “predisposing life events” and “inferred mechanism” components. Regarding the whole formulation, the three pairs of clinicians rated 30 pairs of matched whole formulations and 30 pairs of mismatched whole formulations. In addition, the more experienced clinicians (the first two pairs) rated the two subcomponents; Predisposing life events and Inferred mechanisms. These four judges rated 100 matched and 100 mismatched pairs of formulations for similarity.

### Rater training

The first author trained the other raters. Each rater wrote down a similarity score and then, without changing it, discussed it with the other rater and first author. The training was surprisingly easy, and after training on ten matched and ten mismatched pairs, the rest of the samples were rated independently, without discussion. The discussion between the raters during the calibration period revealed that some differences in rating could be explained by different levels of inference, for example regarding the underlying psychopathology.

## Results

### Completeness

Table 1 shows that 95% of all formulations included information about symptoms. About 83% included at least some information about precipitating stressors. However, one clinician included information about stressors in only 50% of the formulations. Although using some experience-near terms, this clinician used some theoretical constructs and technical language as well, the others managed to avoid this and followed the instructions. Almost all, 99% of the formulations included information about predisposing life events, and 98% included information about an inferred mechanism (See Table 2 for an example of a full case formulation).

**Table 1:** Percentage of case formulations, made by 7 evaluators in the FEST-study, that are deemed complete according to the Case Formulation Content Coding Method

Evaluators	1	2	3	4	5	6	7	Mean
Number of Case Formulations (total *n* = 425)	68	80	32	90	59	37	59	61
	%	%	%	%	%	%	%	%
1. Symptoms and problems	91	99	91	99	90	97	98	95
2. Precipitating stressors or events	50	89	88	87	88	78	96	83
3. Predisposing life events or stressors	98	100	100	99	98	97	100	99
4 Inferred psychological mechanisms	100	100	97	90	98	100	100	98

**Table 2:** Illustrations of full case formulations, by different clinicians (1 and 2), both matched (Patient X) and mismatched (Patient Y)

Patient X Clinician 1	Mostly attached to the mother. The father was authoritarian, somewhat remote, but shared many interests and activities with the patient. A stable, secure childhood. A tendency to have difficulties making decisions since secondary school. Scared by macular bleeding in the eye early in the 20-ies. Indecisive when choosing a career (salesman, artist, author) and reluctant to marry for fear of being limited by all the responsibilities. At the same time guilt feelings for not taking responsibility. Anxiety and depression, self- doubt after giving up a romantic relationship.
Patient X Clinician 2	Grew up in a family with few open conflicts, but father’s authoritarian style seems to have affected the rest of the family. The patient was kind and smart, avoided conflicts. The patient has always had problems making decisions and been bothered by ambivalence with major life decisions like committing to a sweetheart or choosing a career as an artist etc. The romantic relationship was dominated by fear of becoming trapped in a marriage with children where the spouse would be dominant. Chose to move from the partner half a year ago to concentrate on a career as an artist. Ambivalence and anxiety/depressive symptoms for the last 1–2 months after feelings of professional failure. A patient with aggression impairment who easily becomes depressed and anxious when disappointed or irritated. Lots of worries, a strong need for proof of being good enough.
The average similarity in this matched rating (6 raters) was 5.5, range = 4–6.	
Patient Y Clinician 1	Conflicted relationship to a harsh, authoritarian father. A younger brother had a closer relationship to the father. Mother was gentle and flexible and defended the children against the father. Mother became ill and the patient moved to relatives for 6 months when he was 2 years old. Remembers nothing from how he reacted. Lively, somewhat bad tempered. Always jealous of a younger brother. Many friends, restless, active. Intensely in love with a beautiful wife. Two teenage kids. Headache, irritable. Marriage conflicts for many years. But he regards headache and fatigue as non-explainable symptoms. He is like his father, but while his mother resigned, his wife does not. The patient has also symptoms when the burden of responsibilities increases.
The average similarity in this mismatched rating (6 raters) was 2.2, range = 2–3.	

### Reliability of unstructured formulations

The three pairs of clinicians rated 30 randomly selected pairs of matched whole formulations and 30 randomly selected pairs of mismatched formulations. The interrater reliability for the level of similarity for one randomly drawn rater (ICC two-way random, absolute agreement [29]) was excellent, ICC = 0.82 (95% CI 0.75–0.87). The difference in the levels of similarity of same-case pairs versus mismatched pairs across the six evaluators was 4.6 versus 1.9, a mean difference of 2.7 (95% CI 2.1–3.2), (*t* = 10.4, dfs = 57, *p* < 0.001). Each of the six raters rated matched and mismatched pairs significantly different (Tables 3 and 4).

**Table 3:** Mean similarity between raters on matched and mismatched whole case formulations, predisposing events, and Inferred mechanisms, rated on a Likert scale from 1 to 7

	Raters^a^						Mean
	Pair 1		Pair 2		Pair 3		
	1	2	3	4	5	6	
Whole formulation							
Matched	4.6	4.7	4.5	4.2	4.5	4.9	4.6^*^
Mismatched	1.5	2.0	2.1	2.0	1.8	2.0	1.9^*^
Predisposing events							
Matched	5.1	5.0	4.5	4.6	–	–	4.8^**^
Mismatched	2.0	2.0	1.9	2.0	–	–	2.0^**^
Inferred Mechanisms							
Matched	4.3	4.3	3.6	3.5	–	–	3.9^***^
Mismatched	1.8	1.8	1.6	1.7	–	–	1.7^***^

^*^(t = 10.4, df = 57, *p* < 0.00)

^**^(t = 17.3, df = 198, *p* < 0.00)

^***^(t = 15.0, df = 198, *p* < 0.00)

^a^The raters were 6 researchers divided in three pairs: Pair 1 were study clinicians, Pair 2 were experienced clinicians, Pair 3 were inexperienced clinicians

The first four raters were experienced psychodynamic clinicians. The reliability (Intraclass Correlation Coefficient; ICC) of their ratings was 0.79 (95% CI 0.70–0.85). Two raters had no experience in practicing dynamic psychotherapy. The reliability of their ratings was excellent, ICC = 0.91 (95% CI 0.82–0.95).

**Table 4:** Intraclass correlation for similarity ICC two-way random, absolute agreement

	Pair 1 FEST-study clinicians	Pair 2 Experienced clinicians	Pair 3 Resident psychiatrists
Whole formulations (*n* = 60)	0.78	0.82	.91
Predisposing events or stressors (*n* = 200)	0.88	0.85	–
Inferred Mechanisms (*n* = 200)	0.77	0.62	–

### Reliability of two of the formulation components

The two single components in CFCCM requiring more inference: “Predisposing life” (See Table 5.) events and “Inferred mechanism” (See Table 6), were deemed most difficult to formulate and to rate for similarity. The four experienced judges rated 100 matched and 100 mismatched pairs of formulations for similarity. The interrater reliability (ICC) for “Predisposing life events” was 0.82 (95% CI 0.78–0.85). The difference in levels of similarity of matched and mismatched pairs across the four raters was 4.8 versus 2.0. The means are significantly different (t = 17.3, dfs = 198, *p* < 0.000). The mean difference was 2.9 (95% CI 2.5–3.2). Each of the four raters rated matched and mismatched pairs significantly different (Table 4).

**Table 5:** Illustrations of “Predisposing life events”, by different clinicians (1 and 2), both matched (patient X) and mismatched (patient Y)

Patient X Clinician 1:	Mostly attached to the mother. The father was authoritarian, somewhat remote, but shared many interests and activities with the patient. A stable, secure childhood.
Patient X Clinician 2:	Grew up in a family with few open conflicts, but father’s authoritarian style seems to have affected the rest of the family.
The average similarity in this matched rating (4 raters) was 3.75, range 3–6.	
Patient Y Clinician 1:	Conflicted relationship to a harsh, authoritarian father. A younger brother had a closer relationship to the father. Mother was gentle and flexible and defended the children against the father. Mother became ill and the patient moved to relatives for 6 months when he was 2 years old.
The average similarity in this mismatched rating (4 raters) was 2, range 1–4.

**Table 6:** Illustrations of “Inferred mechanism”, by different clinicians (1 and 2), both matched (patient X) and mismatched (patient Y)

Patient X Clinician 1:	A tendency to have difficulties making decisions since secondary school. Scared by macular bleeding in the eye early in the 20-ies. Indecisive when choosing a career (salesman, artist, author) and reluctant to marry for fear of being limited by all the responsibilities. At the same time guilt feelings for not taking responsibility.
Patient X Clinician 2:	The patient was kind and smart, avoided conflicts. The patient has always had problems making decisions and been bothered by ambivalence with major life decisions like committing to a sweetheart or choosing a career as an artist etc. The romantic relationship was dominated by fear of becoming trapped in a marriage with children where the spouse would be dominant. Chose to move from the partner half a year ago to concentrate on a career as an artist. Ambivalence and anxiety/depressive symptoms for the last 1–2 months after feelings of professional failure.
The average similarity on this matched rating (4 raters) was 5.75, ranging from 5 to 6.	
Patient Y Clinician 1:	Lively, somewhat bad tempered. Always jealous of the 1 year younger brother. Many friends, restless, active. Intensely in love with a beautiful wife. Two teenage kids. Headache, irritable. Marriage conflicts for many years. But he regards headache and fatigue as non-explainable symptoms. He is like his father, but while his mother resigned, his wife does not. The patient has also symptoms when the burden of responsibilities increases.
The average similarity on this mismatched rating (4 raters) was 2, ranging from 1 to 4.	

The interrater reliability for “Inferred mechanism” was 0.67 (95% CI 0.61–0.73). The difference in levels of similarity of matched and mismatched pairs across the four raters was 3.9 versus 1.7. The means are significantly different (t = 15.0, dfs = 198, *p* < 0.000). The mean difference was 2.2 (95% CI 1.9–2.5). Each of the four raters rated matched and mismatched pairs significantly different (Table 4).

## Discussion

The main finding in this study is that case-formulations as written by experienced clinicians, without any specific structure or labeling of statements into components, could be rated reliably by experienced as well as less experienced judges. Eells and colleagues [14] also found that novices performed as well as experienced therapists in some comparisons, particularly total formulation quality. They speculated that this could be the result of recent formal training, while experienced clinicians had been out of formal training for years and were overconfident and did not see a need for calibration. It is also possible that inexperienced raters are more “open minded” and read the narratives without so many preconceived theoretical ideas. To the best of our knowledge, this is the first study to rate unstructured formulations reliably. The clinicians in this study were asked to write the formulations using simple experience-near terms, with a minimum of technical language and theoretical jargon. This instruction may have been an important condition that helped achieve the level of agreement that we found. However, the similarity of matched cases was on average only 4.6. That is, the raters thought that only a little more than half of the phrases were similar in meaning. Since our formulations are not based on standard categories, this is to be expected. Furthermore, the formulations are based on a comprehensive semi-structured dynamic interview. From the rich material the clinician must, by inference, select what is essential from what is secondary. Since our knowledge about the causes of mental disorders is limited, selection of what constitutes for example predisposing factors may vary among clinicians. Little is known about how clinicians process clinical information and generate inferences about therapeutic mechanisms and their connections to symptoms and problems. Therapists probably engage in in a great deal of intuitive as well as rational-analytic thinking [30]. The sources of the lower agreement in a number of cases may also be the quality of the dynamic interview or the formulation method rather than the ability of the clinicians to construct reliable narratives. The formulation method in this study was based on Malan’s overall case formulation system. Malan never formally tested the reliability of his method, but DeWitt et al. [31], using Malan’s method, reported that the overall similarity was only 2.9 on matched cases. So far only studies using structured methods report findings of similarity [22, 32] comparable to our study.

To what degree the raters were able to follow the instruction “not to pay attention to descriptive information”, may also have affected the differences in reliability scores. It is probably difficult not to be influenced by contradicting data. This may have inflated our findings. Our findings, however, indicates that highly experienced clinicians can construct reliable formulations. This may not depend on asking clinicians to categorize the information systematically into four components as advocated by Eells [3, 4]. However, by decomposing the formulations into the four components, we could show that both the components, “predisposing life events” and “inferred mechanism” could be rated reliably. It should be noted that for similarity ratings of Inferred mechanisms the lower bound reliability estimate (95% confidence interval) was marginal (0.61). Furthermore, the average degree of similarity for matched cases fell barely at the balance point (4 on the Likert scale from 1 to 7) of equal amounts of overlap and non-overlap. In fact, two of the four evaluators were below this balance point. Mismatched cases were rated well below the balance point. The significant difference in similarity between matched and mismatched cases indicate that psychodynamic formulations as written in this study are to some degree specific to the individual patient, and not some global narrative that apply to most cases.

The inferred mechanism may be the most important part of the psychodynamic case formulation. Eells and colleagues [14], in a study of less experienced clinicians, reported that only 43% inferred a psychological mechanism in their case formulation. Asking clinicians to refer to all components may improve completeness and quality, at least for less experienced clinicians. In this study, almost all case formulations studied had an inferred mechanism. Most inferred mechanisms, however, were a summary of current problems activated by certain stressors, which supposedly were determined by childhood environmental factors, especially relationships to parents and siblings. Concrete experience-near terminology and a relatively low inference level was used in most formulations.

The seven evaluators who wrote the case formulation narratives in this study were experienced psychodynamic clinicians. They had worked together over many years preparing for this psychotherapy study. Hence, they had training in the use of several clinician-rated measures and evaluation of patient self-reports. This may be some of the reasons for the completeness of formulations, and reliability estimates comparable to studies using more structured and standardized methods. Using highly experienced and scientifically trained clinicians to write the formulations may increase internal validity but limit generalizability. Whether our findings can be generalized to narratives written by less experienced clinicians with little or no specific scientific training remains to be seen. To increase the scientific value of psychodynamic case formulations, further studies should examine the reliability and validity of unstructured formulations made by less experienced clinicians.

Clinicians can probably improve the reliability of their formulations by using low-level inferences and avoiding highly speculative inferences. It may be particularly important to ask the patients whether they agree with the formulation. Therapist-patient agreement on the formulation may improve therapeutic alliance and might even be more important than inter-clinician agreement. More generally, clinicians should be aware of heuristics and biases that can lead to unsound judgement.

A major clinical and training implication of these findings is that very experienced clinicians appear able to produce reliable, and thus clinically relevant formulations without elaborate instructions about how to structure the formulation. Further, the use of experience-near, non-theory laden language may facilitate increased clinical utility of a formulation.

## Conclusions

In summary, this study shows that when experienced clinicians freely develop case formulations, they include symptoms and problems, precipitating stressors, predisposing life events, and an inferred mechanism. Additionally, when the clinicians apply a phenomenological approach using a simple experience-near language and minimize technical concepts, other clinicians, both experienced and not, are able to reliably score which formulation is descriptive for which person. This indicates that the case formulations comprise a communicative quality that makes them clinically sound. One may speculate that such case formulations can be helpful when choosing and structuring an intervention. Consequently, they may fill the gap between the symptoms and diagnoses that bring patients to seek help, and the personalized tailored treatment.

## Data Availability

Data from the First Experimental Study of Transference - interpretations (FEST) was used. The data set supporting the results of this article is available from the PI, Per Høglend on reasonable request.

## Abbreviations

CFCCM: Case Formulation Content Coding Method
FEST: First Experimental Study of Transference - interpretations
ICC: Intraclass Correlation Coefficient
